# Purification of Peroxisomes Using a Density Barrier in a Swinging-Bucket Rotor

**DOI:** 10.1100/tsw.2002.837

**Published:** 2002-05-22

**Authors:** John Graham

**Affiliations:** School of Biomolecular Sciences, Liverpool John Moores University, UK

**Keywords:** peroxisomes, light mitochondrial fraction, OptiPrep^TM^, iodixanol, liver, density barrier

## Abstract

In iodixanol, peroxisomes are the densest organelle in the light mitochondrial fraction and are therefore easily separated from the other components (lysosomes, mitochondria, etc.) in a preformed isosmotic continuous gradient. Because of the large difference in density between peroxisomes and the next densest organelle (mitochondria), a density barrier is effective. The resolution of the peroxisomes is far superior than that in sucrose and, unlike in Percoll®, there is no contamination from endoplasmic reticulum.

**METHOD TYPE:** extraction, isolation, purification and separation

**SUB METHOD TYPE:** centrifugation

## INTRODUCTION

Peroxisomes can be purified in iodixanol gradients in high yield (80–90%) with no detectable contamination from any other organelle[1,2,3]. This is a property unique to iodixanol because the densities of other organelles, particularly that of mitochondria (approx. $\rho = 1.14$ g/ml) and endoplasmic reticulum (approx. $\rho = 1.13$ g/ml) are much lower than that of peroxisomes (approx. $\rho = 1.18$ g/ml). In Nycodenz® and metrizamide, mitochondria have a significantly higher density (approx. $\rho = 1.165$ g/ml) than in iodixanol because only the latter can provide an iso-osmotic medium at densities above $\rho = 1.15$–$1.16$ g/ml. The density of peroxisomes on the other hand is relatively little affected by the type of iodinated density gradient medium because of their lack of an osmotic space. In Percoll both peroxisomes and endoplasmic reticulum have the same banding density and these two organelles cannot be resolved.

The protocol below is for the liver from a young adult male rat (wet weight approx. 10 g), other tissues may require a different homogenization technique. It requires a simple iodixanol density barrier in a swinging-bucket rotor (see Note 1). Yields may be lower (approx. 40% of the catalase in the light mitochondrial fraction) than in a continuous gradient but the purity is as high.

## MATERIALS AND EQUIPMENT

OptiPrep™

Working Solution (WS) of 54% (w/v) iodixanol ($\rho$ = 1.291 g/ml): 9 vol of OptiPrep™ + 1 vol of 0.25 M sucrose, 10 mM EDTA, 1% (v/v) ethanol, 200 mM Hepes-NaOH, pH 7.4 (see Note 2)

Homogenization Medium (HM): 0.25 M sucrose, 1 mM EDTA, 0.1% (v/v) ethanol, 20 mM Hepes-NaOH, pH 7.4

High-speed centrifuge with a fixed-angle rotor (30–50 ml tubes)
Ultracentrifuge with swinging-bucket rotor (tube capacity of approx. 17 ml)
Potter-Elvehjem homogenizer (30–40 ml), clearance approx. 0.08 mm
Wall-mounted, high-torque, thyristor-controlled electric motor
Dounce homogenizer (10 ml, loose-fitting, Wheaton Type B)
Syringe with metal cannula for underlayering

## METHOD

1. Make up two dilutions of WS, containing 47% iodixanol ($\rho$ = 1.257 g/ml) and 35% iodixanol (w/v) ($\rho$ = 1.199 g/ml) by mixing WS and HM (8.7 + 1.3 and 6.48 + 3.52 v/v, respectively). Keep these solutions, and carry out all subsequent operations, at 0–4$^{o}$C.
2. Mince the liver very finely with scissors and transfer to a Potter-Elvehjem (Teflon and glass) homogenizer with HM (use 10 ml medium for every 2.5 g tissue). Homogenize using approx. 6 strokes of the pestle (500–700 rpm) (see Note 3).
3. Centrifuge the homogenate at 3000$g_{av}$ in a fixed-angle rotor for 10 min to pellet the nuclei and heavy mitochondria (see Note 4).
4. Centrifuge the supernatant(s) at 17,000$g_{av}$ for 10–15 min to produce a "light mitochondrial pellet".
5. Resuspend this pellet in approx. 8.0 ml of HM (total volume) using a loose-fitting Dounce homogenizer (2–3 strokes of the pestle) and add an equal volume of 47% iodixanol. The refractive index of this suspension should be 1.3782; adjust to this value with the 47% iodixanol or WS if necessary. The density of this suspension is approx. 1.145 g/ml.
6. Transfer approx. 15 ml of the suspension to a 17- to 20-ml tube for a suitable swinging-bucket rotor and underlayer with 1 ml of the 35% iodixanol. Top up with tube with suspension or HM if necessary. Centrifuge at 110,000$g$ for 2 h.
7. Aspirate and discard the majority of the liquid above the peroxisomes which band just above the original interface near the bottom of the tube, then harvest the band of peroxisomes.

**TABLE 1**
**Enzyme Analysis of Fractions**

| Fraction | Catalase[1] | SDH[1] |
|---|---|---|
| Homogenate | 58.6 | nd |
| Light mit pellet | 114.5 | 0.18 |
| Barrier fraction | 1442.8 | 0.02 |

[1] Catalase, in µmoles hydrogen peroxide/min/mg protein, SDH (succinate dehydrogenase), in µmoles succinate oxidized/min/mg protein. nd = not determined.

## ANALYSIS

Iodixanol does not significantly inhibit any enzyme so far tested. Standard spectrophotometric methods (carried out above 340 nm), for measuring organelle enzyme markers can be performed directly on gradient fractions[4]. Protein can also be measured directly by any Coomassie blue-based method[4].

If it is necessary to remove the gradient medium, fractions can be diluted with an equal volume of buffer; pelleted at approx 30,000$g_{av}$ for 10 min and resuspended in a suitable buffer (recoveries are >90%).

A typical enzyme analysis is shown in Table 1 (see Note 5).

## NOTES

1. Other protocols for the isolation of peroxisomes require use either a preformed continuous density gradient[5] or a self-generated gradient in a vertical, or other short sedimentation path length rotor[6]. The simple density barrier technique described in this Protocol Article should provide organelles of similar purity but the yield is likely to be lower than that from the two continuous gradient systems.
2. Strategies for preparing gradient solutions for mammalian tissues are given in Ref. [7]. Protease inhibitors may be included in any or all of the media at the operator's discretion.
3. See Refs. [8,9,10] for more information on homogenization of tissues and cells and differential centrifugation of an homogenate.
4. This pellet may be rehomogenized in HM and the centrifugation repeated.
5. The specific activity of catalase in the barrier fraction represents an approx. 25-fold purification over the homogenate and approx. 37% of the total catalase activity of the light mitochondrial pellet was recovered in the barrier fraction. Barrier techniques, while simple, are inevitably a compromise between purity and yield. Because of the density heterogeneity of all organelles it is impossible to choose a density for the suspending medium which is greater than that all of the mitochondria and less than that of all of the peroxisomes.

## ACKNOWLEDGEMENTS

The author and TheScientific World wish to thank Axis-Shield PoC, AS, Oslo, Norway for their kind permission to adapt OptiPrep™ Application Sheet S10 in the preparation of this Protocol Article.
